# Effects of Hydrogen Peroxide and Sodium Hypochlorite Aging on Properties and Performance of Polyethersulfone Ultrafiltration Membrane

**DOI:** 10.3390/ijerph16203972

**Published:** 2019-10-18

**Authors:** Kai Li, Shu Li, Qian Su, Gang Wen, Tinglin Huang

**Affiliations:** 1 Key Laboratory of Northwest Water Resource, Environment and Ecology, MOE, Xi’an University of Architecture and Technology, Xi’an 710055, China; sisl2018@163.com (S.L.); sunflower190901@sina.com (Q.S.); hitwengang@163.com (G.W.); 2 Shaanxi Key Laboratory of Environmental Engineering, Xi’an University of Architecture and Technology, Xi’an 710055, China

**Keywords:** chemical cleaning, membrane aging, polyethersulfone (PES) ultrafiltration (UF) membrane, hydrogen peroxide (H_2_O_2_), sodium hypochlorite (NaClO)

## Abstract

Chemical reaction of main polymer and additive with oxidative cleaning agents plays an important role in aging of polymeric membrane for water and wastewater treatment. As a green and powerful oxidant, hydrogen peroxide (H_2_O_2_) can achieve good cleaning efficacy under alkaline condition, but its influence on membrane aging was poorly understood. In this study, degradation of polyethersulfone (PES) membrane due to H_2_O_2_ exposure under alkaline condition (pH 9 and 11) was holistically investigated by humic acid (HA) filtration experiments and multiple membrane characterization techniques, with sodium hypochlorite (NaClO) aging examined as a comparison. Membrane permeability and HA retention rate was hardly changed by H_2_O_2_ aging at an exposure dose of 500 g·h/L, whereas NaClO aging led to substantial increase of membrane permeability and significant decrease of retention ability. Meanwhile, H_2_O_2_ aging slightly increased fouling propensity with HA filtration, while NaClO aging resulted in more serious fouling. ATR-FTIR and XPS analysis revealed much less degradation of PES and hydrophilic additive by H_2_O_2_ than that by NaClO, and membrane morphology and surface properties were characterized to explain the variation of filtration performance. Overall, compared with cleaning with NaClO, membrane degradation can be minimized by cleaning with H_2_O_2_.

## 1. Introduction

Ultrafiltration (UF) is an established technology for water and wastewater treatment due to its excellent rejection towards particulates and pathogens, small footprint, and acceptable capital and operation costs [1,2]. However, its wide application is restricted by membrane fouling, which is an inherent drawback of membrane technology [3]. Although membrane fouling can be alleviated by several strategies, such as development of anti-fouling membrane, pretreatment of feed water, and optimization of operation parameters, physically irreversible fouling is still inevitable during long-term operation [4]. Therefore, chemical cleaning is indispensable for the sustainable running of UF system [5]. Acids, bases, oxidants, surfactants, complexing agents, etc. can be used as membrane cleaning agents [6]. Among these cleaning agents, oxidative agents are widely used for membrane cleaning in the water industry because of their high cleaning efficacy for organic and biological fouling, which are major types of fouling in UF for water and wastewater treatment [5,7]. 

Sodium hypochlorite (NaClO) is one of the most commonly used oxidative cleaning agents because of its high efficiency, low cost, and being easy to use [8]. It can disinfect microorganisms adhering to membrane surface, and detach organic and biological foulants from membrane surface by altering their properties [5,9]. However, the reaction of NaClO with organics would generate toxic halogenated by-products, which is an important threat to environmental and public health [10]. More importantly, degradation of membrane materials due to NaClO exposure can alter properties and performance of membrane, and subsequently influence membrane system operation and its lifespan [6,11]. It was found that polyvinylpyrrolidone (PVP), a widely used hydrophilic additive for polyethersulfone (PES) membrane, exhibits high reactivity with NaClO. Surface charge, hydrophilicity, and pore structure might be changed due to oxidation and dislodgement of PVP by NaClO [6,12]. As for the main polymer (i.e., PES) with much higher chemical stability, several studies have reported PES chain scission and/or hydroxylation of PES aromatic rings [13,14,15]. Besides, it was reported that degradation of PES membrane by NaClO was more serious under neutral to weak alkaline conditions, which has been attributed to the formation of hydroxyl radical [14,16,17].

Hydrogen peroxide (H_2_O_2_) is a strong oxidant with a standard reduction potential of 1.78 V, but its reactivity is restricted by a relatively high activation energy barrier [18,19]. Although its application as an oxidative cleaning agent is not as wide as that of NaClO, its effectiveness for permeability recovery has been demonstrated in several studies [20,21]. It was found that H_2_O_2_ cleaning under strong alkaline condition can achieve comparable cleaning efficacy with NaClO for cleaning of UF membrane fouled by humic substances [18]. Moreover, compared with NaClO cleaning, H_2_O_2_ cleaning can avoid the formation of toxic halogenated by-products. Therefore, H_2_O_2_ is regarded as a potential alternative cleaning agent of the widely used NaClO [6]. However, only a few papers have investigated aging of polymeric membrane by H_2_O_2_. Ling et al. [22] and Yu et al. [23] focused on the degradation of polyamide-based reverse osmosis and nanofiltration by H_2_O_2_. With respect to UF membrane, the effects of H_2_O_2_ enhanced backwashing on the mechanical properties and surface functional groups of polyvinylidene fluoride membrane has been examined [24]. In general, aging of UF membrane due to H_2_O_2_ cleaning is poorly understood.

The main purpose of this study was to comprehensively investigate degradation of PES membrane caused by H_2_O_2_ aging, with NaClO aging examined as comparison. PES membrane was soaked in H_2_O_2_ and NaClO solutions with a concentration of 5000 mg/L to accelerate membrane aging, and an exposure dose of 500 g·h/L was selected based on chemical cleaning parameters and frequency generally applied in water and wastewater treatment [6,25]. Membrane permeability, fouling propensity, and retention ability of membranes were examined to evaluate membrane degradation in macroscopic scale. Meanwhile, chemical composition and physicochemical properties of pristine and aged membranes were characterized holistically to elucidate degradation mechanisms and explain the variation in filtration performance.

## 2. Materials and Methods

### 2.1. Chemical Agents

All chemicals and reagents used in this study were analytical grade. Commercial available H_2_O_2_ (~30% wt) and NaClO (~10% wt) were purchased from Tianli Chemical Reagent Co. (Tianjin, China) and Kermel Chemical Reagent Co. (Tianjin, China), respectively. Concentrations of H_2_O_2_ and NaClO solution were determined by permanganate titration method [26] and iodometric titration method [27], respectively, and therefore the reported concentrations were sum of all active species in the solutions. HCl and NaOH were both obtained from Chemical Reagent Co. (Tianjin, China). Humic acid (HA) obtained from Sigma-Aldrich Chemical Co. (St. Louis, MO, USA) was used to evaluate the retention ability and fouling propensity of pristine and aged membranes. Ultrapure water prepared by PURELAB Option-R system (ELGA LabWater, High Wycombe, UK) was used to prepare solutions.

### 2.2. Membranes and Accelerated Aging Procedure

A commercially available flat-sheet PES membrane (UP 150 P, Microdyn-Nadir, Germany) was used in this study, and its main characteristics are listed in Table S1 in the Supplementary Information. According to the manufacturer, the molecular weight cut-offs (MWCOs) of the membrane was 150 kDa. To ensure the removal of preservatives before use, new membranes were soaked in ultrapure water for 36 h and the water was replaced every 12 h. 

In the accelerated aging procedure, H_2_O_2_ and NaClO solutions with a concentration of 5000 mg/L were used as aging solutions, and the membranes were soaked in the aging solutions at ambient temperature (25 ± 2 °C) in the dark for 100 h to obtain an aging intensity (concentration × time, ct) of 500 g·h/L. Aging solutions at pH 9 and 11 were examined because oxidative cleaning agents are usually combined with bases to achieve higher cleaning efficiencies [21,28,29]. The pH of H_2_O_2_ and NaClO solutions were adjusted to 9 and 11 using HCl or NaOH. Meanwhile, to differentiate the influence of H_2_O_2_ and NaClO aging from that of alkaline exposure, NaOH solutions with pH 9 and 11 were also used as aging solutions, and the membrane samples aged in NaOH were denoted as control membrane. To avoid the concentration decay of H_2_O_2_ and NaClO, aging solutions were replaced every 24 h during the experiment. After 100 h soaking in aging solutions, the membranes were rinsed thoroughly and soaked in ultrapure water for another 24 h before characterization and performance evaluation.

### 2.3. Evaluation of Membrane Performance 

Pure water permeability, retention ability, and fouling propensity of pristine and aged membranes were evaluated by filtration experiments conducted in a filtration cell (Amicon 8400, Millipore, MA, USA) in dead-end mode at room temperature (25 ± 2 °C). The UF cell was operated in constant pressure mode with nitrogen gas supplying the driving force, and the trans-membrane pressure (TMP) was kept at 60 kPa. Permeate weight was quantified by an electronic balance connected to a computer and the data were automatically recorded every five seconds.

Fouling propensity and retention ability of pristine and aged membranes were examined by using HA solution as the feed water. The concentration of HA employed in this study was 10 mg/L, and the pH was adjusted to 7.5 ± 0.1 with 0.1 mol/L HCl or NaOH. To simulate the solution chemistry of natural waters, 1 mmol/L of NaHCO_3_, 1 mmol/L of CaCl_2_, and 6 mmol/L of NaCl was added [30]. In each filtration test, 350 mL of HA solution was added to the filtration cell, and filtration was carried out under a TMP of 60 kPa until the permeate volume reached 300mL. Based on initial and final permeate flux as well as permeate volume per unit membrane surface area, the unified membrane fouling index (UMFI) can be calculate to evaluate membrane fouling propensity [16,31]. With respect to retention ability, HA concentrations of feed solution (*C*_f_) and permeate (*C*_p_) were measured in terms of UV absorbance at 254 nm using a UV/Vis spectrophotometer (U-3900, Hitachi, Tokyo, Japan). Membrane retention ability was quantified by the retention rate of HA (Equation (1)).

(1) $$\mathrm{HA}\;\mathrm{rejection}\;\mathrm{rate}=\left(1-\frac{C_{p}}{C_{f}}\right)\times 100$$

### 2.4. Characterization of Membrane Properties

To monitor the changes in functional groups of the membranes, Fourier transform infrared (FTIR) spectrum in the range of 400–4000 cm^−1^ was acquired using an infrared spectrometer (Nicolet iS50, Thermo Scientific, MA, USA). The spectral resolution of the spectrometer was set to be 4 cm^−1^ and each spectrum was an average of 32 scans. X-ray photoelectron spectroscopy (XPS) analysis was performed with a XPS spectrometer (K-Alpha, Thermo Scientific, MA, USA). The deconvolution method of the XPS spectrum was obtained by fitting a Gaussian function. Morphology of membrane surface was observed using a scanning electron microscope (SEM, Quanta 600, FEI, OR, USA). Membrane samples were coated with a thin gold film (3–5 nm) using a precision etching coating system (Model 682, Gatan, USA) before observation. 

Membrane surface charge was measured by an electrokinetic analyzer (SurPASS, Anton Parr, Austria). The electrolyte solution was 1 mM KCl and the channel height was adjusted to 100 ± 5 mm before measurement. The pH of the solution was adjusted from 2.7 to 10.1 by an automatic titrator with a test interval of 0.3. Water contact angle was quantified using a drop shape analyzer (JC2000D4A, Zhongchen, Shanghai, China). 5 μL of ultrapure water was dropped on the membrane surface using a 50 μL glass syringe. After the ultrapure water droplets were dropped on the surface of the membrane for five seconds, the contact angle image between the surface and the water droplets was measured by a light microscope. For each condition, measurements were performed in triplicate using separate pieces of membrane. 

## 3. Results and Discussion

### 3.1. Filtration Performance of Pristine and Aged Membranes 

**Figure 1:**
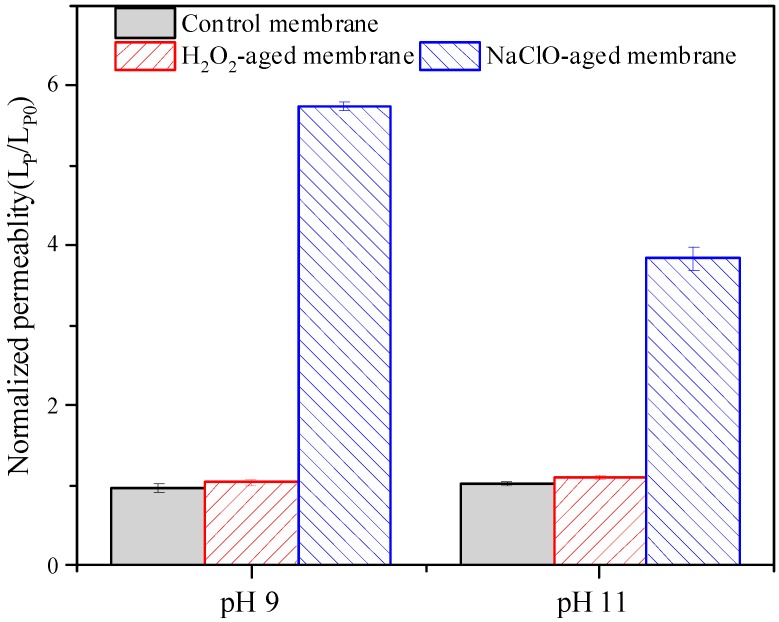
Effects of hydrogen peroxide (H_2_O_2_) and sodium hypochlorite (NaClO) aging on normalized permeability of polyethersulfone (PES) membrane. Control membrane indicates membrane samples aged in NaOH solution with pH 9 or 11, c(H_2_O_2_) = c(NaClO) = 5000 mg/L, t = 100 h, and error bars indicate standard deviation of triplicate samples.

Pure water permeability reflects the intrinsic resistance of membrane to water flow, and it has been widely used as an indicator for the assessment of membrane aging [6,11,12]. In this study, pure water permeability was determined by filtering ultrapure water under a TMP of 60 kPa, and permeability of pristine and aged membranes were denoted as L_p0_ and L_p_, respectively. Permeability of aged membranes normalized to that of pristine membrane (L_p_/L_p0_) are shown in Figure 1. It can be seen that H_2_O_2_ aging at both pH 9 and 11 resulted in insignificant increase of membrane permeability (i.e., less than 10 %). However, permeability of membranes aged by NaClO at pH 9 and 11 were increased to 5.75 and 3.84 times of that of the pristine membrane, respectively. Although the extent of increase varied depending on exposure dose and aging conditions, permeability increase of PES membrane due to NaClO aging has been reported in many studies [13,16,17,32,33,34], and it was commonly attributed to the increase of pore size and porosity [11]. The similar permeability of pristine, control, and H_2_O_2_-aged membranes suggested that pore structure of the PES membrane was not altered obviously by alkaline or H_2_O_2_ under the aging conditions in this work. 

Fouling propensity and retention ability towards organic macromolecules are key performance factors of UF membrane. In this work, HA was used as a model organic foulant to evaluate the retention ability and fouling behavior of pristine and aged PES membranes because it was ubiquitous in natural water and has been identified as a major foulant.

**Figure 2:**
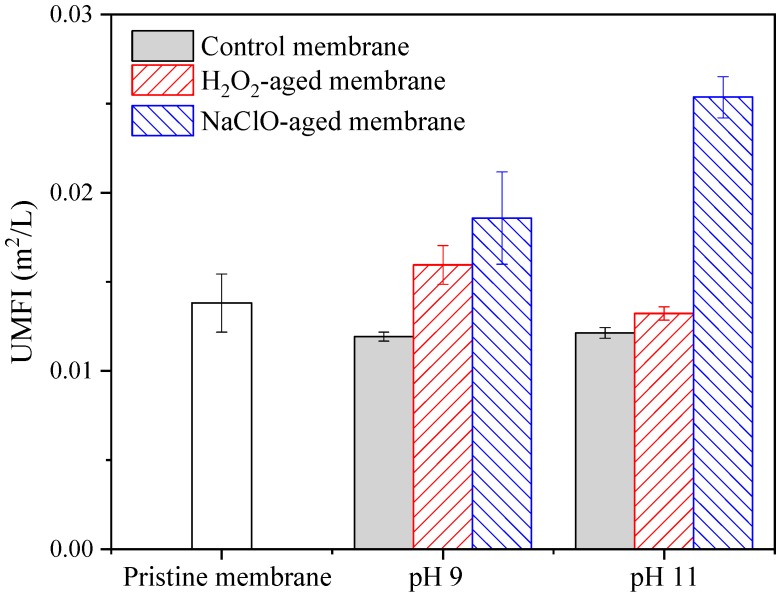
Effects of H_2_O_2_ and NaClO aging on fouling behavior of PES membrane during filtration of humic acid (HA) solution. Control membrane indicates membrane samples aged in NaOH solution with pH 9 or 11, c(H_2_O_2_) = c(NaClO) = 5000 mg/L, t = 100 h, and error bars indicate standard deviation of triplicate samples.

UMFI of pristine and aged membranes for HA filtration are presented in Figure 2. UMFI is a parameter for quantitative description of membrane fouling, and a higher value of UMFI means more serious fouling [31]. Compared with control membrane, UMFI was increased by 33.7% due to H_2_O_2_ aging at pH 9, while the change of UMFI due to H_2_O_2_ aging at pH 11 was less than 10%. As for NaClO aging, UMFI was increased by 55.7% and 109.1 % at pH 9 and 11, respectively. It can be seen that H_2_O_2_ aging resulted in much less increase of fouling propensity compared with NaClO aging. 

**Figure 3:**
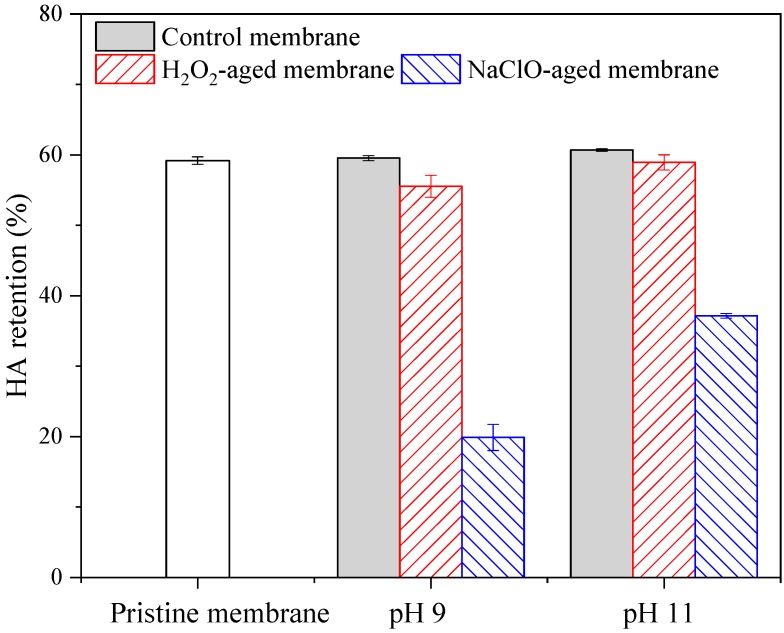
Effects of H_2_O_2_ and NaClO aging on HA retention rate of PES membrane. Control membrane indicates membrane samples aged in NaOH solution with pH 9 or 11, c (H_2_O_2_) = c (NaClO) = 5000 mg/L, t = 100 h, and error bars indicate standard deviation of triplicate samples.

Figure 3 shows HA retention rates of pristine and aged membranes. HA retention rate of pristine membrane was 59.2%, and almost no change in HA retention ability was observed for control membranes. For membranes aged by H_2_O_2_ at pH 9 and 11, the retention rates slightly decreased to 55.5% and 58.9%, respectively. In contrast, the retention rates of membranes aged by NaClO at pH 9 and 11 were significantly decreased to 19.9% and 37.2%, respectively. 

Combining Figure 1, Figure 2 and Figure 3, it can be concluded that degradation of the PES membrane due to NaClO exposure was much more serious than that by H_2_O_2_ exposure under the aging conditions in this work. Considering the comparable efficiency of H_2_O_2_ cleaning with NaClO cleaning [18], H_2_O_2_ is a better oxidative cleaning agent than NaClO because of the minimization of membrane degradation. 

To elucidate the different influences of H_2_O_2_ and NaClO aging on membrane performance, properties of pristine and aged membranes were investigated at both a molecular scale (i.e., functional groups and elements composition) and a microscopic scale (i.e., surface morphology, hydrophilicity, and surface charge).

### 3.2. Chemical Composition of Pristine and Aged Membranes 

**Figure 4:**
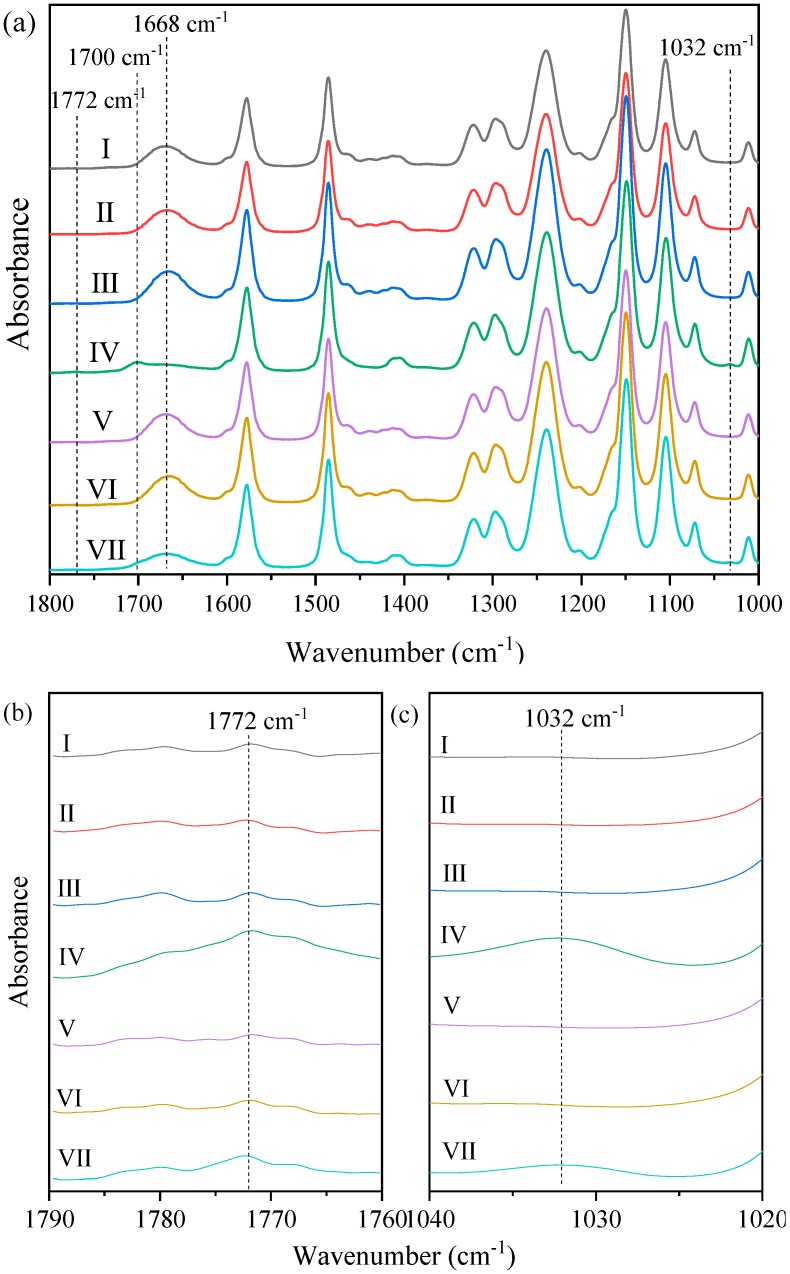
ATR-FTIR spectra of pristine and aged PES membrane: (**a**) 1800–1000 cm^−1^, (**b**) partial enlarged view of 1790–1760 cm^−1^, and (**c**) partial enlarged view of 1040–1020 cm^−1^. (Ⅰ) pristine; (Ⅱ) control (pH 9); (Ⅲ) H_2_O_2_-aged (pH 9); (Ⅳ) NaClO-aged (pH 9); (Ⅴ) control (pH 11); (Ⅵ) H_2_O_2_-aged (pH 11); and (Ⅶ) NaClO-aged (pH 11). Control membrane indicates membrane samples aged in NaOH solution with pH 9 or 11, c(H_2_O_2_) = c(NaClO) = 5000 mg/L, t = 100 h.

The ATR-FTIR spectra of pristine and aged membranes are shown in Figure 4, and the relative absorbance strength at 1772, 1700, 1668, and 1032 cm^−1^ are listed in Table S2 in the Supplementary Information. For pristine membrane, apart from several characteristic peaks of PES (i.e., 1580, 1486, 1320, 1292, 1241, 1150, 1105 cm^−1^) exhibiting high absorption intensity, an obvious band at 1668 cm^−1^ was observed. The band at 1668 cm^−1^ represents the stretching vibration of the amide unit in PVP, and its intensity has been extensively used as an indicator of the content of PVP [16,34,35,36,37]. Similar to previous studies, NaClO aging caused decrease of intensity of the peak at 1668 cm^−1^ due to PVP oxidation /dislodgement, and new peaks at 1700 and 1772 cm^−1^ representing oxidation products of PVP were observed. Meanwhile, a new peak appeared at 1032 cm^−1^ due to NaClO aging. Although there is some controversy concerning the assignment of this peak, it can be ascribed to the formation of sulfonic acid group due to PES chain scission based on the variation of membrane surface charge, which will be discussed in Section 3.3. Compared with NaClO aging at pH 11, NaClO aging at pH 9 resulted in more dramatic changes in peak intensities at 1772, 1700, 1668, and 1032 cm^−1^, suggesting a much higher extent of PVP degradation and PES chain scission at pH 9 than that at pH 11. pH-dependent of PES/PVP degradation by NaClO has been reported in several studies, and it was usually attributed to the formation of hydroxyl radical in NaClO solution due to the coexistence of ClO^−^ and HClO under neutral to weak alkaline conditions [14,38,39]. In contrast, neither decrease of the peak intensity at 1668 cm^−1^ nor appearance of new peaks at 1772, 1700, and 1032 cm^−1^ was observed for control and H_2_O_2_-aged membranes, indicating that PVP oxidation/dislodgement and PES chain scission was insignificant under the aging conditions here.

XPS analysis was conducted to further characterize membrane surface chemical composition of pristine and aged membranes, and atomic percentages of carbon, nitrogen, oxygen, sulfur, and chloride are listed in Table 1. The presence of 4.14% of nitrogen atom further proved the PES membrane used in this study was blended with PVP because there is no nitrogen in pure PES. For membranes aged by NaClO at pH 9 and 11, the atomic percentage of nitrogen decreased to 2.40% and 2.59%, respectively, which was qualitatively consistent with the decrease of peak intensity at 1668 cm^−1^ and demonstrated the dislodgement of PVP. Meanwhile, the incorporation of chloride was observed for NaClO aging, and the atomic percentage of chloride for membrane aged at pH 9 was almost twice of that at 11. Chloride was introduced onto the surface of NaClO-aged membranes in the form of a phenyl chloride group along with the formation of sulfonic acid group during PES chain scission [13,14,40]. The higher percentage of chloride for the membrane aged by NaClO at pH 9 indicated more serious PES chain scission by NaClO aging at pH 9. With respect to H_2_O_2_ aging, although FTIR spectra indicated no peak intensity decrease of the PVP characteristic band (i.e., 1668 cm^−1^), the percentage of nitrogen decreased to 3.27% and 3.80% for pH 9 and 11, respectively. The seemingly contradictory results of ATR-FTIR and XPS analysis can be explained by the different analysis depth of the two techniques, with XPS providing chemical binding information for the top several nanometers of the surface while ATR-FTIR penetrating to a great depth of several micrometers [17,33]. Overall, combining the results of ATR-FTIR and XPS analysis, NaClO aging led to both chain scissions of PES and oxidation/dislodgement of PVP, whereas H_2_O_2_ aging only resulted in partial dislodgement of PVP from a region of very small thickness (Figure S1 in the Supplementary Information). 

### 3.3. Morphology and Surface Properties of Pristine and Aged Membranes

**Figure 5:**
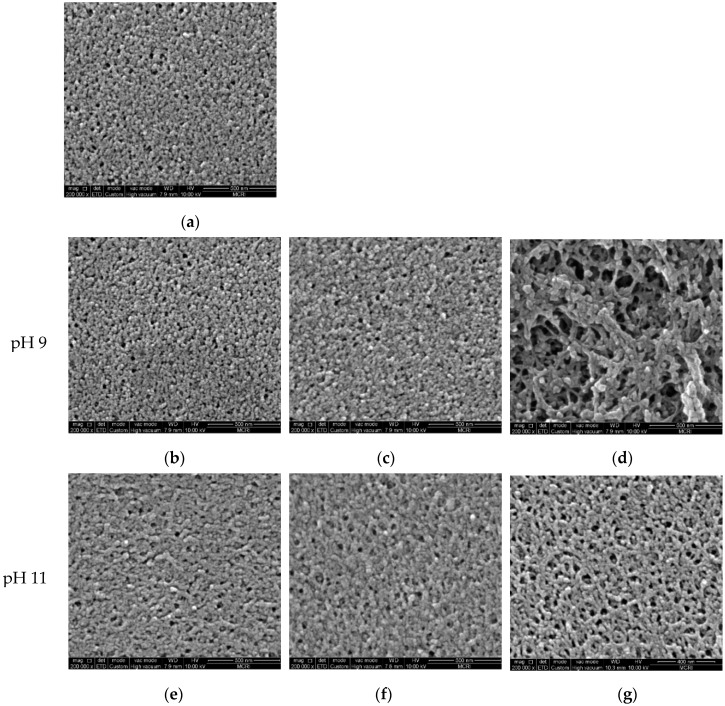
SEM images of pristine and aged PES membrane at the magnification of 200 000: (**a**) pristine; (**b**) control (pH 9); (**c**) H_2_O_2_-aged (pH 9); (**d**) NaClO-aged (pH 9); (**e**) control (pH 11); (**f**) H_2_O_2_-aged (pH 11); and (**g**) NaClO-aged (pH 11). Control membrane indicates membrane samples aged in NaOH solution with pH 9 or 11, c(H_2_O_2_) = c(NaClO) = 5000 mg/L, t = 100 h.

SEM images of pristine and aged PES membranes are shown in Figure 5. It can be seen that membrane surface morphology and pore structure was almost unchanged by alkaline and H_2_O_2_ aging in this study. The partial dislodgement of PVP from membrane surface revealed by XPS analysis did not resulted in visible change in membrane pore structure. This could explain why H_2_O_2_ aging did not result in obvious change in membrane permeability and retention ability. In contrast, NaClO aging led to significant increase of membrane pore size, especially at pH 9 (Figure 5d), which can be attributed to the substantial PES chain scission and PVP degradation [16], and can explain the remarkable increase in membrane permeability and decrease in HA retention rate. 

**Figure 6:**
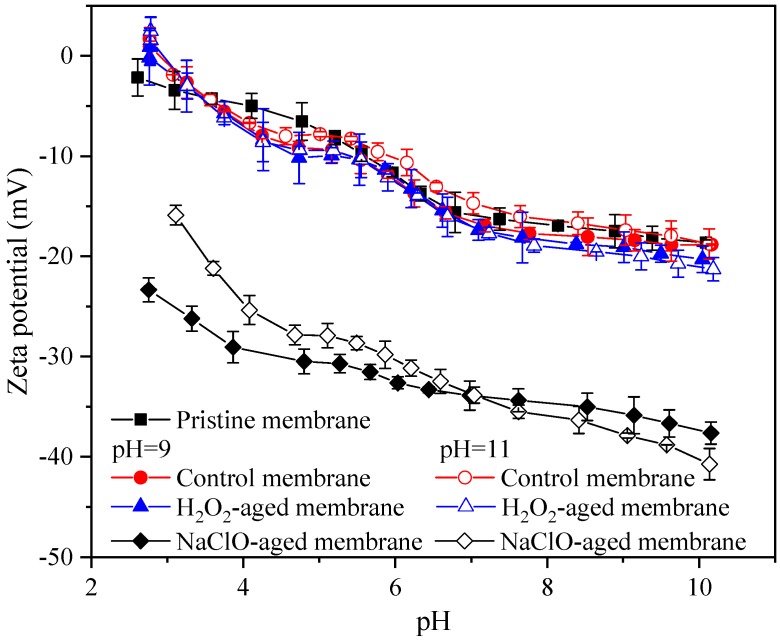
Effects of H_2_O_2_ and NaClO aging on pH dependence of the zeta potential of PES membrane. Control membrane indicates membrane samples aged in NaOH solution with pH 9 or 11, c(H_2_O_2_) = c(NaClO) = 5000 mg/L, t = 100 h, and error bars indicate standard deviation of triplicate samples.

Figure 6 shows the variation of zeta potentials of pristine and aged membranes in the pH range from of 2.7–10.1. The pristine membrane exhibited an isoelectric point (IEP) of about 2.7, and the negative charge increased with solution pH, indicating the presence of weak acid groups such as carboxylic acid groups [14,40]. The zeta potentials of control and H_2_O_2_-aged membranes were similar with that of pristine membrane, suggesting that alkaline and H_2_O_2_ aging did not alter ionizable functional groups on membrane surface, which was consistent with the result of ATR-FTIR. However, the IEP of membranes aged by NaClO disappeared and negative charge at solution pH around 3 increased substantially, indicating the formation of strong acid functional groups. Therefore, the peak formed at 1032 cm^−1^ in ATR-FTIR analysis (Figure 4c) was assigned to sulfonic acid group [14,40]. At solution pH around 3, the negative charge of the membrane aged by NaClO at pH 9 was higher than that aged by NaClO at pH 11, which was consistent with the more serious PES chain scission demonstrated by ATR-FTIR and XPS analysis. Moreover, compared with membrane aged by NaClO at pH 9, the membrane aged by NaClO at pH 11 exhibited steeper increase of negative charge with the increase of solution pH. The difference suggested that more carboxylic acid groups generated by PVP oxidation present on membrane surface, which could be ascribed to less dislodgement of oxidation products at pH 11. 

**Figure 7:**
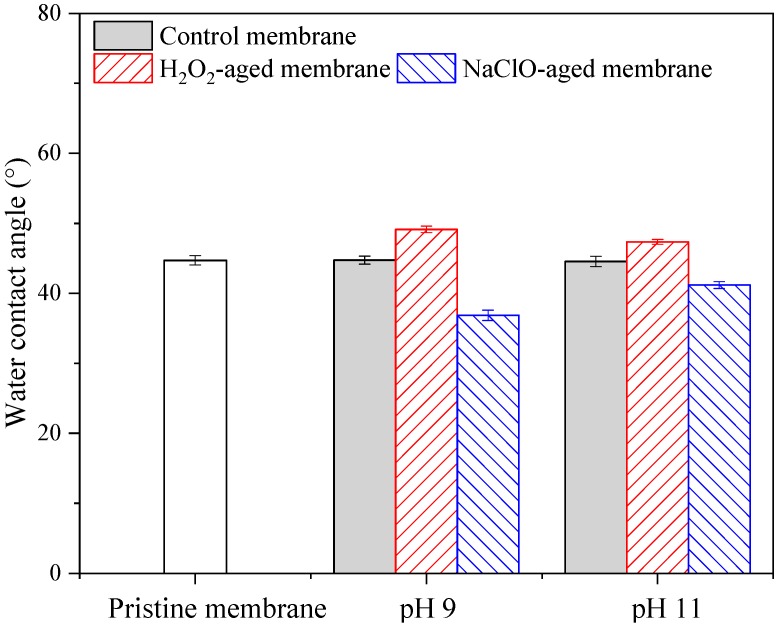
Effects of H_2_O_2_ and NaClO aging on pure water contact angle of PES membrane. Control membrane indicates membrane samples aged in NaOH solution with pH 9 or 11, c(H_2_O_2_) = c(NaClO) = 5000 mg/L, t = 100 h, and error bars indicate standard deviation of triplicate samples.

The water contact angles of pristine and aged membranes are shown in Figure 7. Water contact angle of pristine membrane was 44.7°, which was lower than pure PES material probably due to blending with PVP [33]. H_2_O_2_ aging resulted in some increase of water contact angle, which might be ascribed to the dislodgement of some PVP from membrane surface as revealed by XPS analysis (Table 1). The increase of water contact angle can explain the slight increase of fouling propensity of H_2_O_2_-aged membrane (Figure 2). However, NaClO aging at pH 9 and 11 decreased the water contact angle to 36.9° and 41.2°, respectively. Although dislodgement of hydrophilic additive (i.e., PVP) generally leads to decrease of hydrophilicity and increase of water contact angle, the decrease of water contact angle due to NaClO aging has been reported in several previous studies and was attributed to the increase of membrane pore size and capillary effect [7,36]. In fact, the variation of water contact angle was decided by several factors, including loss of hydrophilic additive, increase of pore size, and increase of negative charge due to PES and PVP degradation. The decrease of water contact angle here did not lead to the alleviation of hydrophobic interactions. Moreover, increase of membrane pore size might also result in the change of fouling type and increase of fouling propensity [41]. Therefore, although NaClO aging caused a decrease of water contact angle and increase of surface negative charge, fouling propensity of membrane aged by NaClO was increased.

## 4. Conclusions

In this study, effects of H_2_O_2_ and NaClO exposure under alkaline condition (pH 9 and 11) on filtration performance and physicochemical properties of PES membrane were comprehensively investigated. H_2_O_2_ aging did not result in obvious change in membrane permeability and retention ability, whereas NaClO aging led to remarkable increase of permeability and substantial decrease of retention ability. Meanwhile, H_2_O_2_ aging slightly increased fouling propensity during HA filtration, while NaClO aging resulted in more serious fouling. ATR-FTIR and XPS analysis suggested that H_2_O_2_ aging resulted in partial dislodgement of PVP from membrane surface, but the main polymer was not degraded. Therefore, membrane hydrophilicity was slightly decreased, while membrane morphology and surface charge remained unchanged. In contrast, NaClO aging not only resulted in substantial oxidation and dislodgement of PVP, but also caused chain scission of PES, leading to significant increase in membrane pore size and surface charge. Overall, the degree of membrane degradation caused by H_2_O_2_ exposure was much lower than that by NaClO exposure, and aging of PES membrane due to chemical cleaning can be minimized by using H_2_O_2_ as cleaning agent.

## Figures and Tables

**Table 1:** Atomic percentage of elements (at%) of pristine and aged PES membranes. Control membrane indicates membrane samples aged in NaOH solution with pH 9 or 11, c(H_2_O_2_) = c(NaClO) = 5000 mg/L, t = 100 h.

Element.	Pristine	pH 9			pH 11		
		Control	H_2_O_2_-Aged	NaClO-Aged	Control	H_2_O_2_-Aged	NaClO-Aged
C	73.72	74.47	73.66	71.15	74.57	74.51	72.49
N	4.14	4.57	3.27	2.40	4.30	3.80	2.59
O	17.65	16.65	17.71	18.88	16.35	16.65	18.51
S	4.50	4.31	5.35	6.26	4.79	5.03	5.72
Cl	-	-	-	1.30	-	-	0.69

## Supplementary Materials

The following are available online at https://www.mdpi.com/1660-4601/16/20/3972/s1, Figure S1. Schematic diagram of PES and PVP degradation by H_2_O_2_ and NaClO. For H_2_O_2_ aging, no PES degradation was detected by FTIR, XPS or zeta potential measurement, while XPS indicated partial loss of PVP from membrane surface with a very small thickness (several nm). For NaClO aging, the formation of sulfonic acid group revealed by FTIR (1032 cm^−1^), the incorporation of chloride revealed by XPS, and the increase of membrane surface negative charge suggested chain scission of part PES, while two degradation products of PVP, i.e., succinimide and carboxyl group due to opening of the pyrrolidone ring, was proved by FTIR (1700/1772 cm^−1^) and zeta potential measurement, respectively. Phenyl chloride and carboxyl group were not detected by FTIR probably due to their relatively low abundance. Table S1: Characteristics of PES membrane (UP150 P) provided by the manufacturer. Table S2: The relative absorbance strength of pristine and aged PES membranes at 1772, 1700, 1668 and 1032 cm^−1^ by normalizing to the absorbance at 1240 cm^−1^. Control membrane indicates membrane samples aged in NaOH solution with pH 9 or 11, c(H_2_O_2_) = c(NaClO) = 5000 mg/L, t = 100 h.
