# Supplementary material for: Effects of Hydrogen Peroxide and Sodium Hypochlorite Aging on Properties and Performance of Polyethersulfone Ultrafiltration Membrane
Source: Int J Environ Res Public Health. 2019 Oct 18;16(20):3972. doi: 10.3390/ijerph16203972 (PMC6843545; doi:10.3390/ijerph16203972)
Supplement: Supplementary file 1 [file ijerph-16-03972-s001.pdf]

Supplementary Information

Effects of Hydrogen Peroxide and Sodium Hypochlorite Aging on Properties and Performance of Polyethersulfone Ultrafiltration Membrane

Kai Li [1,2,*], Shu Li [1,2], Qian Su [1,2], Gang Wen [1,2] and Tinglin Huang [1,2,*]

[1] Key Laboratory of Northwest Water Resource, Environment and Ecology, MOE, Xi'an University of Architecture and Technology, Xi'an 710055, China; likai@xauat.edu.cn (K. L.), sisl2018@163.com (S. L.), sunflower190901@sina.com (Q. S.), hitwengang@163.com (G. W.), huangtinglin@xauat.edu.cn (T. H.)

[2] Shaanxi Key Laboratory of Environmental Engineering, Xi'an University of Architecture and Technology, Xi'an 710055, China

[*] Correspondence: likai@xauat.edu.cn (K.L.); huangtinglin@xauat.edu.cn (T.H.); Tel.: +86-029-8220-7886 (K.L.); +86-029-8220-1038 (T.H.)

Table S1. Characteristics of PES membrane (UP150 P) provided by the manufacturer.

MWCO (kDa)	Permeability [a] (L/(m^2·h))	Retention rate for Dextran-2000 [a] (%)	Membrane thickness (μm)	Operation condition	
				pH	Temperature (℃)
150	>200	91-97	210-250	0-14	5-95

a: test condition: 0.7 bar, 20 ℃, stirring rate 700 rpm.

Table S2. The relative absorbance strength of pristine and aged PES membranes at 1772, 1700, 1668 and 1032 cm^{-1} by normalizing to the absorbance at 1240 cm^{-1}. Control membrane indicates membrane samples aged in NaOH solution with pH 9 or 11, c(H_2O_2) = c(NaClO)= 5000 mg/L, t=100 h.

Wavenumber (cm^{-1})	Pristine	pH 9			pH 11		
		Control	H_2O_2-aged	NaClO-aged	Control	H_2O_2-aged	NaClO-aged
1772	0.018	0.016	0.015	0.024	0.016	0.015	0.019
1700	0.061	0.058	0.051	0.092	0.060	0.052	0.069
1668	0.201	0.212	0.219	0.076	0.219	0.213	0.130
1032	0.067	0.059	0.054	0.079	0.055	0.057	0.071

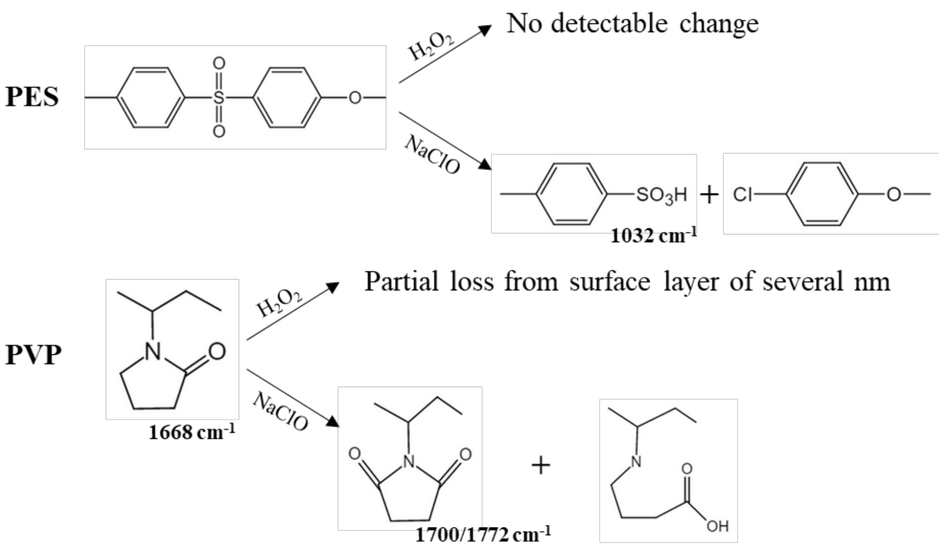

Figure S1. Schematic diagram of PES and PVP degradation by H_2O_2 and NaClO. For H_2O_2 aging, no PES degradation was detected by FTIR, XPS or zeta potential measurement, while XPS indicated partial loss of PVP from membrane surface with a very small thickness (several nm). For NaClO aging, the formation of sulfonic acid group revealed by FTIR (1032 cm^{-1}), the incorporation of chloride revealed by XPS, and the increase of membrane surface negative charge suggested chain scission of part PES, while two degradation products of PVP, i.e., succinimide and carboxyl group due to opening of the pyrrolidone ring, was proved by FTIR (1700/1772 cm^{-1}) and zeta potential measurement, respectively. Phenyl chloride and carboxyl group were not detected by FTIR probably due to their relatively low abundance.
